# Editing of the ethylene biosynthesis gene in carnation using CRISPR-Cas9 ribonucleoprotein complex

**DOI:** 10.1186/s13007-024-01143-0

**Published:** 2024-02-02

**Authors:** Oluwaseun Suleimon Adedeji, Aung Htay Naing, Hyunhee Kang, Junping Xu, Mi Young Chung, Chang Kil Kim

**Affiliations:** 1 Department of Horticulture, Kyungpook National University, Daegu, 41566 South Korea; 2 Life Science and Technology School, Lingnan Normal University, Zhanjiang, 524048 China; 3 Department of Agricultural Education, Sunchon National University, Suncheon, South Korea

**Keywords:** Calli, CRISPR/Cas9, Ethylene biosynthesis genes, Indel patterns, In vitro cleavage, Protoplast

## Abstract

The study aimed to edit ethylene (ET) biosynthesis genes [*1-aminocyclopropane-1-carboxylic acid (ACC) synthetase 1* (*ACS1*) and *ACC oxidase 1* (*ACO1*)] in carnation using the CRISPR/Cas9 ribonucleoprotein (RNP) complex system. Initially, the conserved regions of the target genes (*ACS1* and *ACO1*) were validated for the generation of different single guide RNAs (sgRNAs), followed by the use of an in vitro cleavage assay to confirm the ability of the sgRNAs to cleave the target genes specifically. The in vitro cleavage assay revealed that the sgRNAs were highly effective in cleaving their respective target regions. The complex of sgRNA: Cas9 was directly delivered into the carnation protoplast, and the target genes in the protoplast were deep-sequenced. The results revealed that the sgRNAs were applicable for editing the ET biosynthesis genes, as the mutation frequency ranged from 8.8 to 10.8% for *ACO1* and 0.2–58.5% for *ACS1*. When sequencing the target genes in the callus derived from the protoplasts transformed with sgRNA: Cas9, different indel patterns (+ 1, − 1, and − 8 bp) in *ACO1* and (− 1, + 1, and + 11) in *ACS1* were identified. This study highlighted the potential application of CRISPR/Cas9 RNP complex system in facilitating precise gene editing for ET biosynthesis in carnation.

**Supplementary Information:**

The online version contains supplementary material available at 10.1186/s13007-024-01143-0.

## Introduction

Carnations (*Dianthus caryophyllus*) possess fragile petals and vibrant hues, which has contributed to their widespread popularity in the floricultural industry. These flowers often take centre stage during momentous occasions due to their symbolism of affection, admiration, and memorial sentiments. The cultivation of carnations has undergone remarkable expansion in both its global market value and production volume. The global market value of carnations has already attained an impressive 2.47 billion USD and is anticipated to rise to 2.9 billion USD by 2024 [1, 2]. However, the delicate nature of these floral arrangements often poses a challenge in maintaining their freshness and longevity. Ethylene (ET) is a naturally occurring plant hormone synthesized from the transcription of specific ET biosynthesis genes *aminocyclopropane-1-carboxylic synthase* (*ACS*) and *aminocyclopropane-1-carboxylic oxidase* (*ACO*). ET plays a crucial role in determining the vase life of the flowers as it acts as a positive regulator of flower senescence [3–9].

The flower market continues to exhibit a growing demand for carnations with an extended vase life. Although ET inhibitors such as silver thiosulfate complex (STS) [5, 10], aminoethoxyvinylglycine (AVG) [11], nano-silver [5, 8, 12], and sodium nitroprusside [13] can be employed to delay the senescence of carnation flowers, major concerns have arisen regarding potential human health risks due to chemical exposure and potential environmental contamination resulting from the disposal of these chemical compounds. Therefore, the application of ET inhibitors does not appear to be a promising approach for extending the longevity of carnation flowers.

CRISPR/Cas9 system has emerged as the technology of choice for editing unwanted genes due to its simplicity, specificity, efficiency, and versatility. Petal senescence-related genes have been successfully edited in morning glory, rose and petunia using CRISPR/Cas9 system to extend flower longevity [9, 14–16]. Xu et al. [9, 16] reported that editing of ET biosynthesis genes (*ACO1*, *ACO3*, and *ACO4*) in petunia significantly reduced ET production and improved flower longevity, and the edited genes are also stably transmitted to subsequent generations. In carnation, ET biosynthesis genes encoding ACS and ACO enzymes have been identified as *DcACS1*, *DcACS2*, *DcACS3,* and *ACO1* [17–19]. whereas *DCACS1* and *DcACO1* were most abundant in petals and gynoecium, notably inducing ET production and flower senescence. Previous studies also have indicated that petal senescence in carnation is regulated by transcriptional regulation of *DcACS1* and *DcACO1*, and the application of ET inhibitors delayed petal senescence by suppressing the expression levels of *DcACS1* and *DcACO1*, as well as ET production [8, 12, 13]. The editing of the ET biosynthesis genes (*DcACS1* and *DcACO1*) in carnation using the CRISPR/Cas9 system will be a promising approach for achieving a permanent reduction of ET production and improvement of flower longevity. Generally, gene-edited mutants were generated through the application of *Agrobacterium*-mediated transformation; however, this approach often leads to an off-target mutation, a major challenge in genome editing. An alternative approach involves the direct delivery of preassembled CRISPR/Cas9 ribonucleoproteins (RNPs) into protoplasts. As Svitashev et al. [20] demonstrated, this strategy significantly successfully reduced off-target mutations. These RNPs rapidly initiate cleavage at chromosomal target sites upon transfection and are only transiently present in plant cells prior to degradation by proteases and nucleases [21, 22]. This swift breakdown mechanism within cells can potentially diminish mosaicism and the occurrence of off-target effects during the regeneration process of entire plants [22]. Therefore, in this study, we edited the ET biosynthesis genes (*DcACS1* and *DcACO1*) in carnation by delivering preassembled CRISPR/Cas9 ribonucleoproteins (RNPs) into the protoplast.

## Materials and methods

### Plant materials

In vitro regenerated carnation plantlets (*Dianthus caryophyllus* cv. Scarlet) obtained from Gyeongsang National University were subcultured on Murashige & Skoog (MS) medium supplemented with 30 g/L sucrose, 1.0 g/L activated charcoal, and 8.0 g/L plant agar. The culture bottles were placed in a culture room set at a temperature of 25 °C, a photoperiod of 16 h (70 mmol m^−2^ s^−1^), and a relative humidity (RH) of 70%. Subculturing of the plantlets was performed every 6 weeks using the same fresh medium.

### Designation of single guide RNA (sgRNA)

**Fig. 1:**
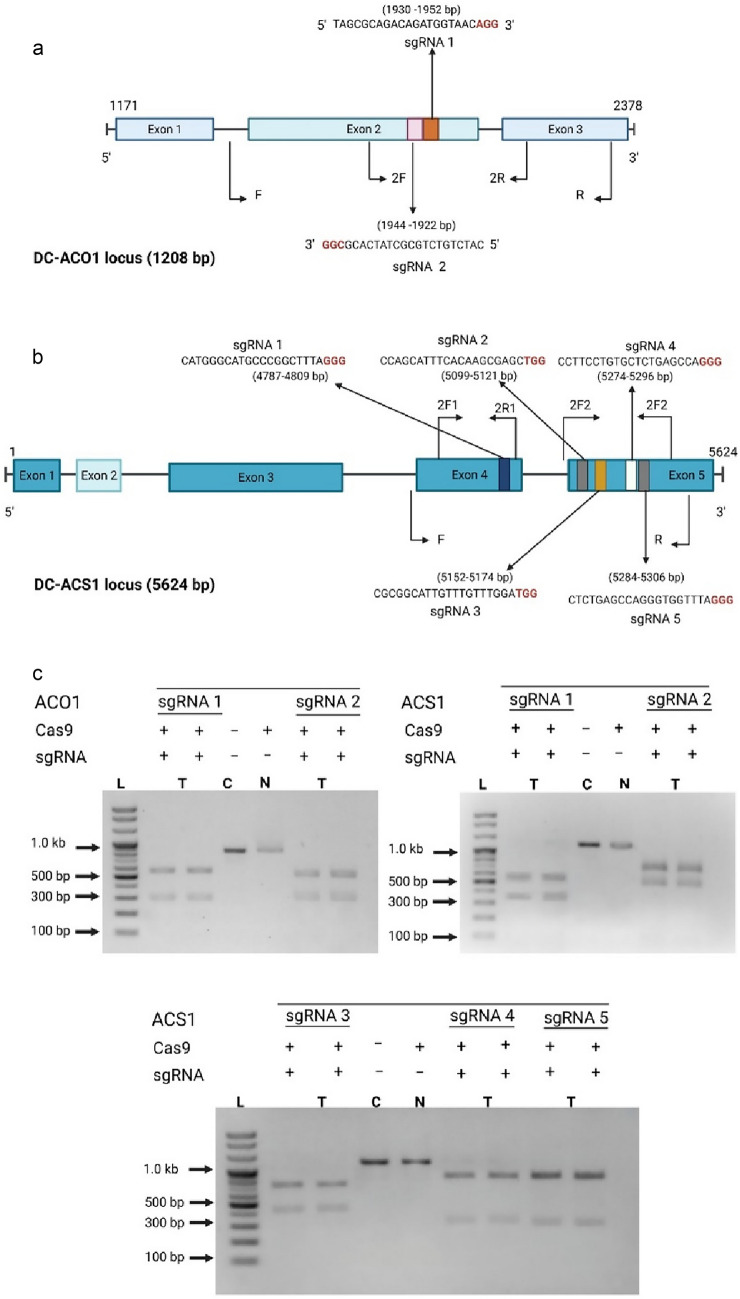
Schematic diagram of the target gene locus. **a**. Target locus of the two gRNAs designed for the *DcACO1* gene. **b**. Target locus of the five gRNAs designed for the *DcACS1* gene. **c**. Cleavage assay for CRISPR RNPs in *DcACO1* and *DcACS1* gene. Lanes L, DNA ladder, T, treated with Cas9 and gRNA, *C* positive control, and *N* negative control

To edit the ET biosynthesis genes (*DcACO1* and *DcACS1*) in carnation cv. Scarlet, their exon regions were determined based on the complete sequences of *DcACO1* (AB042320.1) and *DcACS1* (AB605175.1). Five sgRNAs originating from the fourth and the fifth exons of *ACS1*, as well as two sgRNAs from the second exon of *ACO1,* were individually designed using the CRISPR RGEN tool (http://rgenome.net/) Additional file 1: Fig. S1, following the approach outlined by Park et al. (2015). The exon regions and sequences of the sgRNAs were illustrated in (Fig. 1a, b) and detailed in Additional file 1: Table S1. To ensure precise gene editing, we meticulously selected sgRNAs with no more than two nucleotide mismatches and higher out-of-frame scores. This stringent selection process aimed to guarantee both high specificity and maximum knockout efficiency in the coding regions of the carnation *DcACO1* and *DcACS1* genes. Readily available recombinant Cas9 protein and sgRNAs were purchased from ToolGen, Inc. (Seoul, South Korea).

### In vitro cleavage assay

Genomic DNA was extracted from young carnation leaves using the HiGene Genomic DNA Prep Kit (Biofact, South Korea), following the manufacturer’s instructions. The target regions of *DcACS1* and *DcACO1* were amplified using Phusion polymerase (Thermo Fisher Scientific, Inc. Vilnius, Lithuania) and specific primer pairs (*DcACO1* F: 5ʹ AACATCTCCGAGGTCCCTGA 3ʹ R: 5ʹ TGAGATGAGATGAGAGTGGCG 3, DcACS1 F: 5’ ATGACATGCAGATTCCGCGA 3’ R: 5ʹ ACCCTTCCACGGGTTACAAA 3ʹ). The PCR products were then purified using an Expin PCR SV kit (GeneAll, Seoul, South Korea). Subsequently, the purified PCR products (150 ng) were digested using purified Cas9 protein (1 μg) and sgRNA (0.5 μg) in a 20 μL reaction mixture that included 1 μL of 10 × NEB 3.1 buffer (NEB) (New England Biolabs Inc. USA), 1 μL of 10X bovine serum albumin (BSA) (Elpis Biotech, South Korea.), and nuclease-free water (NFW). The reaction was incubated for 1 h at 37 °C, followed by an additional incubation with RNase A for 15 min at 37 °C. To halt the digestion, 1 μL of STOP solution (30% glycerol, 1.2% SDS, 250 mM EDTA (pH 8.0) was added to the reaction mixture and incubated for a further 15 min at 37 °C before analysis on a 2% agarose gel. The schematic flow chart of the in vitro cleavage assay is depicted in Additional file 1: Fig. S2.

### Protoplast isolation

Protoplasts were isolated from the leaves of in vitro plants, as performed by [1]. Briefly, leaf segments (approximately 1.0 g fresh weight) were finely chopped into 0.5 mm pieces using a sharp razor blade. These 0.5 mm pieces were then placed in a falcon tube containing a mixture of 10 ml of cell and protoplast washing (CPW) solution and 0.5 M mannitol. To induce cell wall plasmolysis, the falcon tube was incubated in a shaking incubator at 30 rpm for 1 h at 25 °C. The plasmolyzed cells were then digested in 10 ml of CPW solution comprising 1.2% cellulase, 0.1% macerozyme, 0.1% bovine serum, and 0.5 M mannitol for 6 h at 25 °C. The resulting protoplast was collected by passing through a 100 µm nylon mesh. The collected protoplast was allowed to float on a CPW solution containing 3.0% sucrose, and 2 ml of W5 was pipetted gently onto the sucrose solution. Next, the sucrose solution and washing solution were centrifuged at 180 ×*g* for 10 min to examine the formation of a viable protoplast floating band at interphases of the sucrose and W5 layers. The viable protoplast solution was collected into a fresh tube using a Pasteur pipette. This solution was then diluted 10 times with W5 solution and centrifuged for 5 min at 125 ×*g*. The centrifugation was repeated three times. Finally, the protoplasts were resuspended in W5 solution and allowed to rest for 30 min at 4 °C prior to the transfection process.

### Delivery of sgRNA: CRISPR/Cas9 RNP complex into protoplast

The sgRNAs (25 µg) of *ACS1* and *ACO1* were separately mixed with 25 µg of cas9 protein in 2 µl of NEB 3.1 and sterile distilled water to obtain a ribonuclease protein (RNP) complex with a final volume of 20 µl. Control samples were established with only Cas9 protein without sgRNA. The RNP complex was then incubated for 15 min at 25 °C in the dark. Following this, 100 µl (2 × 10^5^/ml) protoplasts were gently mixed with the RNP complex, and 120 µl of PEG solution (25% PEG 4000, 0.1 M CaCl_2_, and 0.4 M mannitol) was added to the solution (RNP and protoplasts) for delivery of RNP complex into protoplasts. The mixture was then incubated for 15 min at 25 °C in the dark and washed three times at 5 min intervals using 1 ml, 2 ml, and 5 ml of washing solution. The mixture was then centrifuged at 125 ×*g* for 3 min. Finally, the protoplasts were resuspended in 1 ml of W5 solution in the dark for 24 h. The protoplast transfection was performed following the optimal protocol of Adedeji et al. [1].

### DNA Extraction and targeted deep sequencing

Genomic DNA was extracted from both protoplasts transfected with either Cas9 alone or with RNPs, using a DNeasy plant mini kit (Qiagen, Hilden, Germany), following the manufacturer’s instructions. The gene loci of *DcACO1* and *DcACS1* were amplified using a Phusion High-Fidelity PCR kit (Thermo Fisher Scientific, Inc. Vilnius, Lithuania) along with the primer pairs indicated in Additional file 1: Table S2. The amplified PCR products were sequenced using an Illumina MiSeq (Macrogen, Seoul, South Korea). The resulting data were analysed using the Cas analyzer (http://www.rgenome.net/cas-analyzer/#!) as performed by [23]. The sgRNAs with the highest indel frequency for each gene were selected for further investigation.

### Delivery of selected sgRNA: CRISPR/Cas9 RNP complex into protoplast and callus induction

sgRNA1 (for *DcACS1*) and sgRNA2 (for *DcACO1*) were selected for editing the respective genes in the protoplasts. The sgRNAs were selected based on their high indel frequency and distinctive patterns, as determined from the analysis mentioned above. The protoplasts (at a concentration of 1.0 × 10^5^ protoplasts/ml) that were transfected with the RNP complex (*ACS1*: sgRNA, and *ACO1*: sgRNA) were cultured in petri dishes (60 × 15 mm) containing 2.5 ml of MS liquid media with 0.5 M mannitol, 1% sucrose, 0.1 g/L casein hydrolysate (CH), and different concentrations of plant growth regulators (PGRs) (Additional file 1: Table S3). The petri dishes were sealed with parafilm and kept in the dark at 25 °C. After 12 days of culture, 1 ml of fresh culture media was added to the dishes every 7 days. After 25 days of culture, the osmotic potential was gradually increased by reducing the mannitol concentration by 20% each week, and the cultures were subjected to low light conditions. After 6 weeks of culture, the formation of microcalli (0.5 mm in size) was observed, and the microcalli were transferred onto MS solid media containing 2% sucrose, 0.2 mg/L 2,4-D, and 0.25% gelrite for microcalli proliferation. Next, 3 week-old calli were transferred to a regeneration medium for shoot induction.

### Analysis of indel patterns and protein sequence in *ACS1*- and *ACO1*- edited callus lines

For identifying indel patterns of *ACS1* and *ACO1* in the callus derived from the protoplasts transfected with the RNP complex (*ACS1*: sgRNA and *ACO1*: sgRNA), genomic DNA was extracted from single green calli (18 single cell lines), and target regions of the *DcACO1* and *DcASC1* genes were amplified as described above. The resulting amplicons were sequenced using the Sanger sequencing tool (Macrogen, Seoul, South Korea). The indel patterns were then assessed using DECODR V.3.0 software, while the protein sequences were analyzed through the website www.bioinformatics.org/sms2/translate.

### Statistical analysis

SPSS Statistics software version 25 (SPSS Inc., Chicago, IL, USA) was used to analyze the experimental data. All experiments were replicated three times, and data were presented as means of three experiments. Significant differences among treatments were determined at *p* < 0.05 based on the least significant difference test.

## Results

### Generation of sgRNAs and validation of their effi cacy in in vitro cleavage

First, the exon regions of the *DcAC01* and *DcACS1* in carnation cv. Scarlet was verified using Sanger sequencing, resulting in five exon regions in *DcACS1* and three exon regions in *DcACO1* (Fig. 1a, b). Five distinct sgRNAs (sgRNA1-5) were designed from fourth and fifth exon regions of *DcACS1*, and two different sgRNAs (sgRNA1 and 2) were designed from second exon region of *DcACO1*. To ensure the selection of the most suitable sgRNAs, we performed a thorough homology search against the *Dianthus caryophyllus* reference genome using Cas-Designer from RGEN tools [24]. Our criteria included confirming no more than two nucleotide mismatches in the selected sgRNAs.

To assess the effectiveness of the designed sgRNAs in editing the target genes, we performed an in vitro cleavage assay for each sgRNA. This assay employed Cas9 protein and in vitro transcribed sgRNAs. As expected, both sgRNA 1 and sgRNA 2 cleaved the 852 bp of the *DcACO1* PCR amplicon into approximately 548 and 304 bp (Fig. 1c and Table 1). However, in *DcACS1*, the sgRNAs exhibited differential cleavage patterns on the 1217 bp of the *DcACS1* PCR amplicon, depending on the specific sgRNA used. For sgRNA1, the cleavage resulted in approximately 579 and 397 bp fragments; for sgRNA2, it was approximately 728 and 489 bp fragments; for sgRNA3, it was approximately 802 and 415 bp fragments; for sgRNA4, it was approximately 883 and 334 bp fragments, and for sgRNA5 it was approximately 893 and 324 bp fragments (Fig. 1c and Table 1). The results clearly demonstrate that all the designed sgRNAs were highly effective in cleaving their respective target regions.

**Table 1:** In vitro cleavage efficiency of sgRNAs targeting the *DcACO1* and *DcACS1* gene loci

Gene	sgRNAs	PCR Amplicon (bp)	Cleaved fragments (bp)
*DcACO1*	1	852	548 and 304
	2	852	548 and 304
*DcACS1*	1	1217	579 and 397
	2	1217	728 and 489
	3	1217	802 and 415
	4	1217	883 and 334
	5	1217	893 and 324

### Assessment of indel percentages and patterns in *ACS1* and *ACO1*

**Fig. 2:**
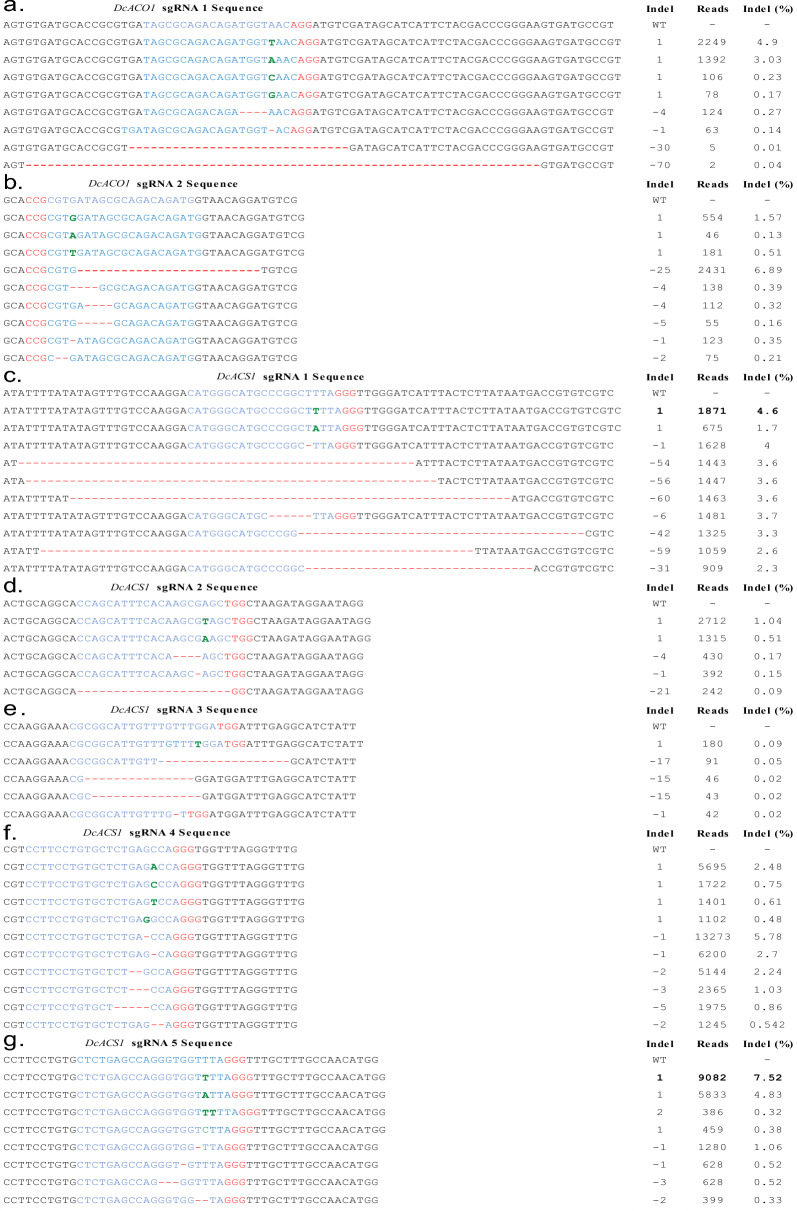
Targeted deep sequencing analysis of transformed carnation protoplasts for *DcACO1* and *DcACS1* genes showing the most frequent mutation patterns. Blue- Target sequence, red- PAM sequence, green- insertions

To access insertion/deletion (indel) percentages and patterns in *ACS1* and *ACO1*, CRISPR/Cas9 RNP complex (Cas9 and each sgRNA) was introduced into carnation protoplasts using the PEG-mediated transfection method. This targeted deep sequencing was performed to examine the indel percentages and patterns at the target regions. The results revealed that each sgRNA can induce a range of indel percentages and patterns, with variations depending on the specific sgRNA used, as depicted in (Fig. 2). Specifically, sgRNA1 induced + 1 bp, − 1 bp, − 4 bp at target site of *ACO1*, with the highest indel percentages observed for + 1 bp and  −1 bp (4.9% and 3.03%) (Fig. 2a). Similarly, sgRNA2 also induced diverse indel patterns (+ 1, − 1, − 2, − 4, − 5, and − 25 bp) at the target site of ACO1*,* with the highest indel percentage (6.89%) observed for − 25 bp (Fig. 2 b)*.* Similar diverse indel patterns were also observed in the target region of *ACS1* when the different sgRNAs were employed (Fig. 2c–g). Interestingly, indel patterns were even observed outside the target regions of *ACS1* and *ACO1*; *DcACO1* sgRNA 1 induced a − 70 bp outside the target region, while *DcACS1* sgRNA 1, 2, 3 and 5 induced− 60, − 21, − 17 and − 9 bp indels outside the target region, respectively. Large insertions of + 29 and + 35 bp were observed in sgRNA4, while + 27 and + 29 bp were also observed in sgRNA5 (Fig. 2 and Additional file 1: Fig. S3). In contrast, no indel was detected in the non-transfected protoplast samples (wild type; WT). These results provide compelling evidence of the direct delivery of the RNP complex to carnation protoplasts. The observed total indel percentages ranged from 8.8 to 10.8% for *DcACO1* and from 0.2 to 58.5% for *DcACS1* (Table 2). After evaluating multiple sgRNAs, sgRNA 2, which exhibited an indel percentage of 10.8%, was chosen as the most promising candidate for editing *DcACO1*. Similarly, sgRNA 1, with an indel percentage of 58.5%, was selected for editing *DcACS1*.

**Table 2:** Mutation rates in the transformed protoplasts, quantified by targeted deep sequencing

Gene	sgRNAs	Total reads	Indel	Indel frequency (%)
Control	–	32,223	0	0
*DcACO1*	1	45,946	4044	8.80
	2	35,360	3809	10.80
Control	–	221,036	0	0
*DcACS1*	1	40,405	23,637	58.50
	2	259,713	6337	2.44
	3	201,000	402	0.20
	4	229,641	44,252	19.27
	5	120,779	21,547	17.84

### Callus induction from the protoplasts transfected with selected sgRNA: CRISPR/Cas9 RNP complex

**Fig. 3:**
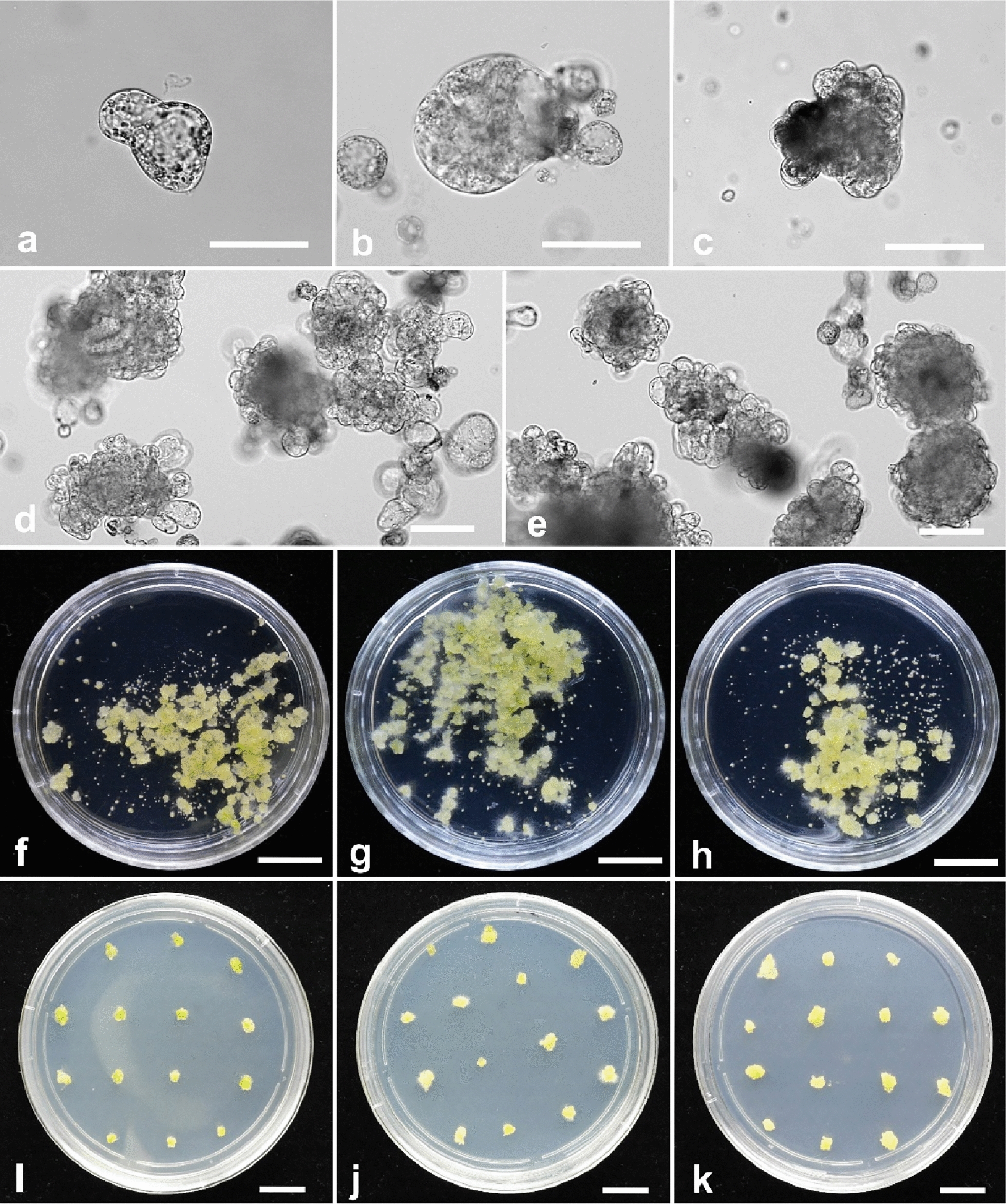
Protoplast developmental stages. **a**. First cell division at 5 days (× 20). **b**. multi-cellular division at 10 days (× 20). **c**. Microcalli formation at 14 days (× 20). **d**. *DcACO1* transfected protoplast at 21 days old (× 10). **e**. *DcACS1* transfected protoplast at 21 days old (× 10). Calli induction from protoplast-derived micro calli. **f**. non-transfected protoplast. **g**. *DcACO1* transfected protoplast. **h**. *DcACS1* transfected protoplast. Protoplast-derived calli on shoot induction media. **i**. non-transfected protoplast. **j**. *DcACO1* transfected protoplast. **k**. *DcACS1* transfected protoplast

The RNP complexes [Cas9: sgRNA1 (for *DcACS1*) and Cas9: sgRNA2 (for *DcACO1*)] were introduced into the carnation protoplasts, and the protoplasts were cultured in MS liquid media with 0.5 M mannitol, 1% sucrose, 0.1 g/L casein hydrolysate (CH), and different concentrations of PGRs. The developmental stages, colony formation, and calli formation from the protoplasts significantly varied depending on the PGRs used (Table 3). The protoplasts cultured in media supplemented with only zeatin or NAA died within 6 days of culture. Those cultured in media supplemented with either 0.5 mg/L or 1.0 mg/L 2,4-D alone formed microcolonies but halted their development in the third week of culture. However, when protoplasts were cultured in media containing a combination of distinct cytokinins paired with auxin or a mix of two different auxins, they progressed into multicellular divisions and eventually developed into calli. In media enriched with 1.0 mg/L zeatin and 1.0 mg/L 2,4-D, or alternatively, with 0.5 mg/L NAA and 0.5 mg/L 2,4-D, the initial protoplast cell division became evident within 5–7 days of cultivation (Fig. 3a). Subsequently, multi-cellular division and the formation of microcalli were observed at the 10-day and 14-day marks (Fig. 3b, c). The progression of microcalli development from transfected protoplasts of *DcACO1* and *DcACS1* can be visualized in Fig. 3d, e. The highest division frequency and several calli formations were observed in 0.5 mg/L 2,4-D with 0.5 mg/L NAA, as well as in 1.0 mg/L zeatin with 1.0 mg/L 2,4-D. The transfected protoplast was cultured in media supplemented with 1.0 mg/L zeatin with 1.0 mg/L 2,4-D and subsequently transferred to MS media with 0.2 mg/L 2,4-D to promote calli proliferation and subsequently on shoot induction media supplemented with different combination of PGRs (Fig. 3f–k).

**Table 3:** Effect of different PGR combinations and concentrations on protoplast culture

No	PGR (mg/l)	Division freq. (%)	Colony formation	Days to colony formation	No. of calli/10^5^ protoplast
1	1.0 Zeatin	−	−	−	–
2	0.5 2,4-D	63.56 ± 0.76^d^	+ + +	9	−
3	1.0 2,4-D	77.12 ± 1.42^ab^	+ + +	9	−
4	0.5 NAA	-	−	−	−
5	0.5 NAA 0.5 2,4-D	82.40 ± 2.10^a^	+ + + +	8	984 ± 3.12^a^
6	0.5 Zeatin, 1.0 2,4-D	66.83 ± 0.87^c^	+ +	12	423 ± 2.11^c^
7	1.0 Zeatin, 1.0 2,4-D	78.66 ± 1.24^a^	+ + + +	10	1032 ± 4.13^a^
8	2.0 Zeatin, 1.0 2,4-D	72.8 ± 2.33^bc^	+ + +	10	765 ± 6.00^b^
9	0.5 BA, 1.0 2,4-D	64.80 ± 1.52^c^	+	12	272 ± 8.15^d^
10	1.0 BA, 1.0 2,4-D	72.39 ± 0.91^bc^	+ + +	12	474 ± 6.40^c^
11	0.5 TDZ, 1.0 2,4-D	66.12 ± 0.80^c^	+ +	12	551 ± 3.128^c^
12	1.0 TDZ, 1.0 2,4-D	68.40 ± 1.12^c^	+ + +	12	763 ± 4.77^b^
13	1.0 Zeatin, 1.0 NAA	63.80 ± 3.02^d^	+	10	−
14	1.0 Zeatin, 3.0 NAA	67.5 ± 0.67^c^	+	10	−
15	1.0 Zeatin, 5.0 NAA	74.3 ± 1.17^b^	+	10	−

*The data represent the mean of three replicates per treatment. Means with the same letter are not significantly different by Duncan’s multiple range test (DMRT, p < 0.05). Division frequency, colony formation (no. of cells that divided more than three times), and the number of calli were calculated in 8 days, 12 days, and 9 weeks after protoplast culture, respectively. —Not observed, + Poor, +  + Moderate, +  +  + Good, +  +  +  + Excellent

### Assessment of indel percentages and patterns in *ACS1* and *ACO1* of protoplast-derived callus

**Fig. 4:**
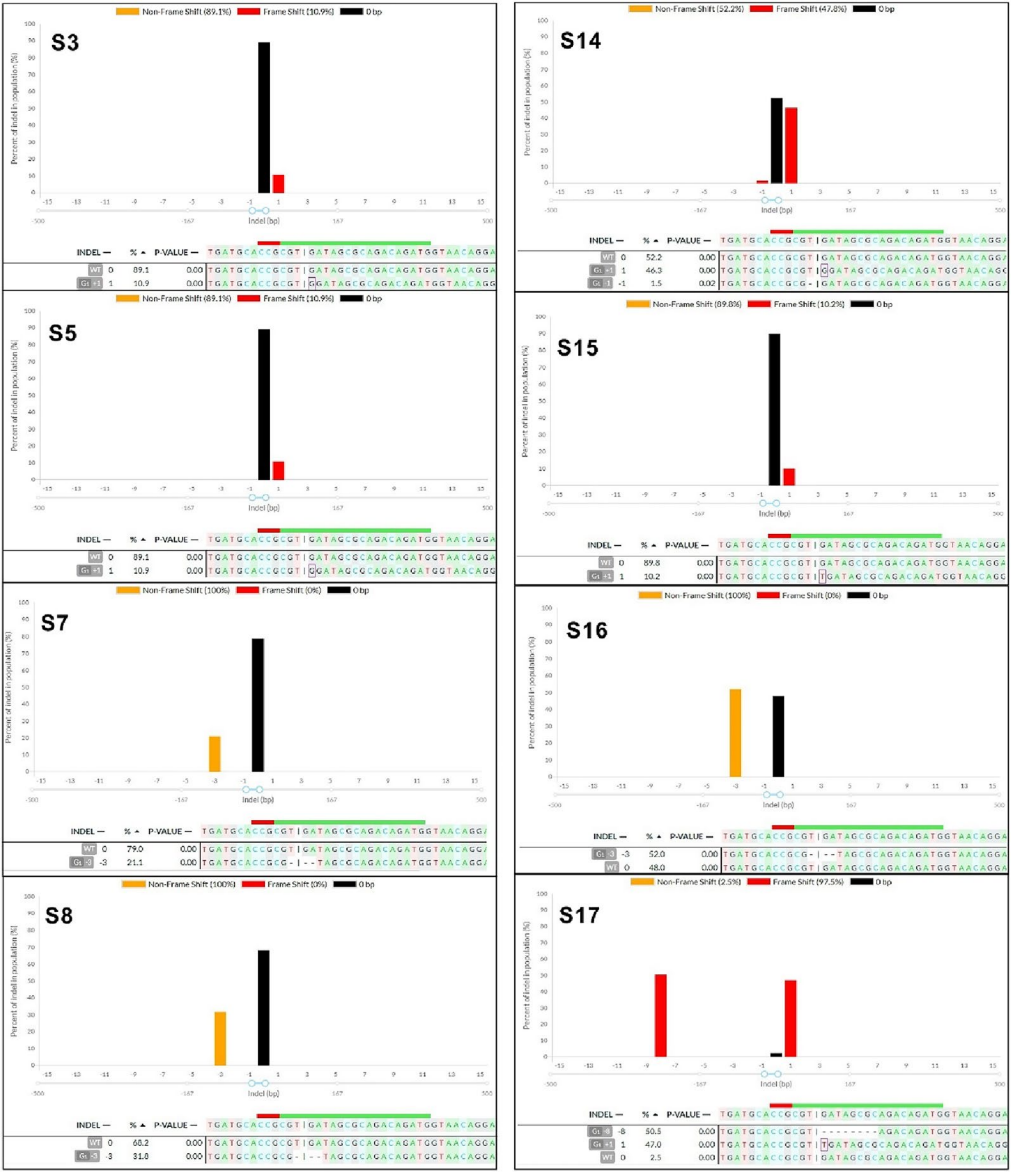
Illustration of indel patterns in the calli derived from carnation protoplasts transfected with sgRNA1: *DcACO1*. These results were generated by analysing the Sanger sequencing results with DECODR software. The top panels display the graphs for the indel distribution rate. The bottom panel shows the list of sequences as alignments, indel patterns, and percentages (%). Insertion (highlighted with purple rectangles) and deletion [minus sign (−)] of mutations are shown in alignments. A 20 bp target and 3 bp PAM site are depicted with green and red lines, respectively

**Fig. 5:**
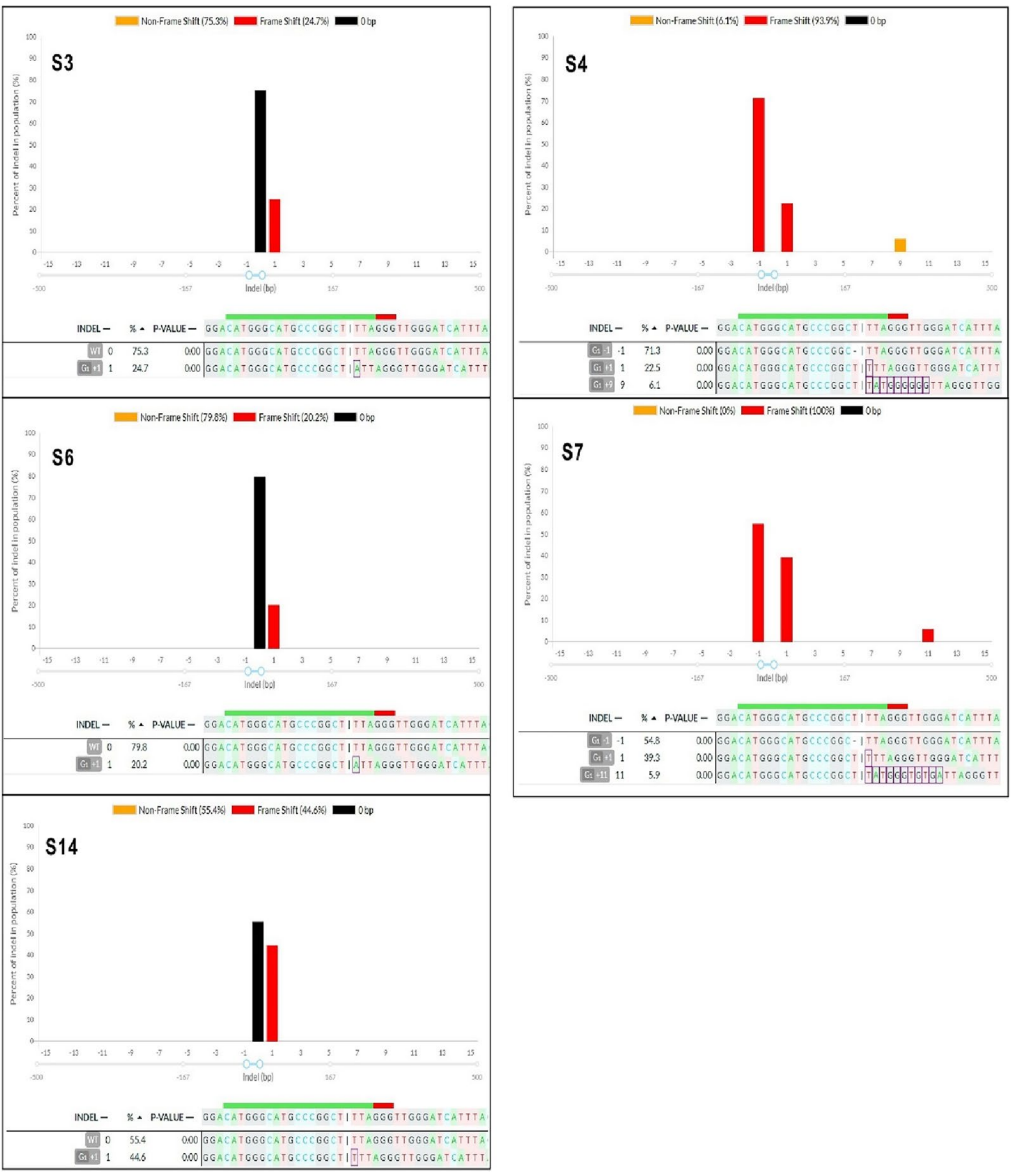
Illustration of indel patterns in the calli derived from carnation protoplasts transfected with sgRNA1: *DcACS1*. These results with DECODR software. The top panels display the graphs for the indel distribution rate. The 23 bottom panel shows the list of sequences as alignments, indel patterns, and percentages (%). Insertion (highlighted with purple rectangles) and deletion [minus sign (−)] of mutations are shown in alignments. A 20 bp target and 3 bp PAM site are depicted with green and red lines, respectively

From the above experiment, we randomly selected 18 single calli derived from protoplasts, which were transfected with the RNP complexes [Cas9: sgRNA1 (for *DcACS1*) and Cas9: sgRNA2 (for *DcACO1*)]. The results of the Sanger sequencing coupled with DECODR online CRISPR analysis software showed that 8 out of 18 samples for *DcACO1 and* 5 out of 18 samples for *DcACS1* were mutants, indicating the indel percentages of 44.4% in *DcACO1* and 27.8% in *DcACS1* (Table 4). Additionally, in *DcACO1*, 75% of the callus lines showed monoallelic mutations, while 25% exhibited biallelic mutations. Remarkably, no instances of triallelic mutations were observed in this context. In contrast, among the 18 samples assessed for *DcACS1*, 60% indicated monoallelic mutations, while the remaining 40% were indicative of triallelic mutations. Biallelic mutations, however, were not detected within this set of samples. Furthermore, different indel patterns were also observed within the mutant samples. Notably, samples 14 (S14), 16 (S16), and 17 (S17) exhibited the highest indel percentages, accounting for 47.8% indel with a + 1 bp (G) insertion, 52.0% with a − 3 bp deletion, and a remarkable 97.5% indel of a + 1 bp (T) insertion combined with a − 8 bp deletion at the designated *DcACO1* target site respectively. Similarly, S5 exhibited a + 1 bp insertion, and S8 presented a − 3 bp deletion, with indel frequencies of 46.9% and 31.8% respectively, both at the target site of *DcACO1* (Fig. 4). In the case of *DcACS1*, S6 and S7 exhibited the highest indel percentages, both at 100%, characterized by a 71.3% indel with a − 1 bp (T) deletion, 22.5% with a + 1 bp (T) insertion, and 6.1% with a + 9 bp insertion, as well as 54.8% indel with a –1 bp (T) deletion, 39.3% with a + 1 bp (T) insertion, and 5.1% with + 11 bp insertion, respectively, at the specified target site (Fig. 5). Similarly, S3, S4, and S14 showcased indels of + 1 (A) insertion at 24.7%, + 1 (A) insertion at 20.2%, and + 1 (T) insertion at 44.6%, respectively (Fig. 5). Additionally, we identified frameshift mutations that would disrupt the reading frame and result in the complete loss of function for both genes. Specifically, we found that the alleles with + 1, − 1, and − 8 bp mutations in *DcACO1* and − 1, + 1, and + 11 bp mutations in *DcACS1* were frameshift mutations. Additionally, we observed in-frame mutations at the target sites, such as a − 3 bp deletion in S7, S8, and S16 (*DcACO1*) and a + 9 bp insertion in S17 (*DcACS1*). The in-frame mutations observed in the *DcACO1* and *DcACS1* genes hold the potential to modify or partially impair their protein function. These predictions were made using the online tool https://bioinformatics.org/sms2/translate.html, and the corresponding results can be found in Tables 5, 6.

**Table 4:** Percentages of callus lines found with different mutation types in the target sequence

Targeted gene	Total samples # of samples analyzed	# of samples with indels (%)	# of samples with monoallelic mutation (%)	# of samples with biallelic mutation (%)	# of samples with triallelic mutation (%)
*DcACO1*	18	8 (44.4)	6 (75)	2 (25)	0 (0)
*DcACS1*	18	5 (27.8)	3 (60)	0 (0)	2 (40)

**Table 5:**
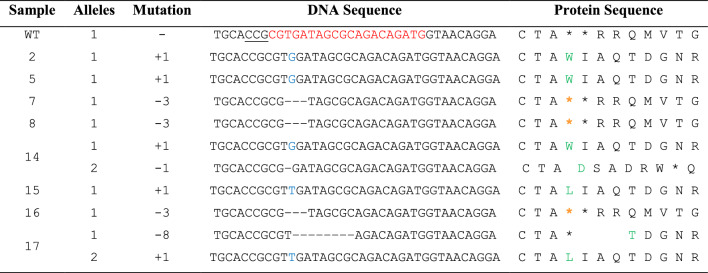
Illustration of protein sequence changes in the calli derived from carnation protoplast transfected with sgRNA2: *DcACO1*

^*^Underlined—PAM sequences (NGG), Red—target sequence, Blue—inserted nucleotide, minus sign (−)—deleted nucleotide, Green—start of amino acid change, Grey—Inserted amino acid, Orange—deleted amino acid

**Table 6:**
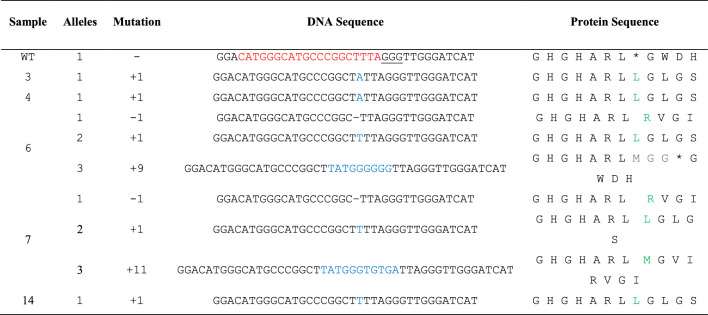
Illustration of protein sequence changes in the calli derived from carnation protoplast transfected with sgRNA1: *DcACS1*

^*^Underlined—PAM sequences (NGG), Red—target sequence, Blue—inserted nucleotide, minus sign (−)—deleted nucleotide, Green—start of amino acid change, Grey—Inserted amino acid, Orange—deleted amino acid

## Discussion

Floral senescence in carnations is positively associated with increased ethylene production, which is caused by the transcriptional activation of ET biosynthesis genes like *ACO* and *ACS* [17–19]. [9, 16] successfully delayed floral senescence in petunia by editing ET biosynthesis genes (*ACO1*, *ACO3*, and *ACO4*) using the CRISPR/Cas9 tool. In this study, we attempted to edit the ET biosynthesis genes (*ACS1* and *ACO1*) in carnation using the CRISPR/Cas9 tool. Although *Agrobacterium*-mediated transformation technique has been employed for editing target genes with CRISPR/Cas9 tool, this method carries the potential for off-target mutations [20, 25]. In addition, this method uses selection marker genes for screening of mutants, which has raised concerns among consumers. Instead, CRISPR/Cas9 RNP complex tool, which does not involve the use of selection marker genes, has been increasingly favoured in recent years for editing target genes to reduce the potential for off-target mutation and to address consumers’ concerns. Success in CRISPR/Cas9 RNP complex-mediated editing of target genes depends on various factors, including sgRNA design, transient expression efficiency of Cas9 and sgRNA complex in protoplast, and the formation of protoplast-derived callus [26, 27].

In this study, we designed two sgRNAs targeting *DcACO1* and five sgRNAs targeting *DcACS1*, and their editing efficiencies were assessed through in vitro DNA cleavage assays and a protoplast transient assay. All employed sgRNAs exhibited editing activity. However, the results of targeted deep sequencing showed that indel frequencies of two sgRNAs targeting *DcACO1* ranged from 8.8 to 10.8% at the *DcACO1* locus and of five sgRNAs targeting *DcACS1* ranged from 0.2 to 58.5% at *DcACS1* locus within carnation protoplasts. These findings are consistent with a previous study by [28], who also found varied indel patterns at cleavage sites when using different sgRNAs for editing the *nitrate reductase* gene in petunia. The variation in the indel frequencies among the tested sgRNAs may be attributed to several factors, including the secondary structure of the sgRNA molecule, a factor that can influence its affinity for Cas9 binding and its overall stability [29]. One more reason for this phenomenon was attributed to discernible double-strand breaks (DSBs) and the subsequent engagement of non-homologous end-joining (NHEJ) repair mechanisms [30].

The short insertions (+ 1 or + 2 bp) or deletions (− 1 to − 6 bp) were observed across all the sgRNAs targeted for the *DcACO1* and *DcACS1* gene. In addition, a large deletion of − 70 bp at the *DcACO1* locus was also observed when *DcACO1* sgRNA 1 was employed. Similarly, *DcACS1* sgRNA 1, 2, 3, and 5 induced the deletions of − 60, − 21, − 17, and − 29 bp (Fig. 2). [22] also reported a large deletion of − 223 bp in *Arabidopsis*. We observed that the editing efficiency of sgRNA2 and sgRNA3 targeted for *DcACS1* was comparatively low, at 2.44% and 0.2%, respectively. This could be attributed to various factors, including the targeted loci's chromatin states, unwanted hairpin structures, or potential unidentified elements [31, 32]. In a study done by [31], the chromatin state of the target locus was a significant determinant of the editing efficiency, and the presence of hairpin structures could reduce the editing efficiency by up to 50%. We tested multiple sgRNAs for *DcACO1* and *DcACS1* and selected sgRNA2 and sgRNA1, respectively, based on NGS results for further experiments. In the case of *DcACO1*, our analysis revealed that sgRNA1 and sgRNA2 had similar indel frequencies, however, sgRNA2 produced a large − 25 bp deletion with 6.89% indel frequency, which could lead to a complete loss of function in the *DcACO1* gene loci. Subsequently, sgRNA1 was selected for *DcACS1*, as it has a high indel frequency (58.5%) and is less likely to produce off-target effects.

Media composition, PGR supplementation, and culture conditions need to be considered to develop an effective method for protoplast culture [7] because their effects on protoplast culture vary depending on genotypes and plant ages [33–36]. We observed significant differences in the developmental stages, colony formation, and calli formation of protoplasts when exposed to various combinations of PGRs. Protoplasts cultured solely in media containing zeatin or NAA experienced cell death within 6 days. Conversely, when cultured with either 0.5 mg/L or 1.0 mg/L of 2,4-D alone, the protoplasts formed microcolonies, but their development halted by the third week of culture. This cessation may be attributed to inadequate cell wall regeneration, a vital process for sustained mitotic division. Poorly developed cell walls can result in abnormal mitosis and prevent further cell division [7]. Moreover, we noticed a delay of 2 days in the cell division stages of transformed protoplasts compared to non-transformed protoplasts, which could be attributed to the stress experienced during the transfection process. Additionally, we observed that the proliferation and regeneration of protoplasts were influenced by the specific type and combination of PGRs used; this is consistent with findings in other plant species [33, 37].

[38] edited the *F3H* gene in petunia using CRISPR/Cas9 RNP complex to modify flower colour, whereas they obtained 11.9% of mutants. Similarly, [39] also edited *CCD7* and *CCD8* in tomato using CRISPR/Cas9 RNP complex, with an editing efficiency (26%) for two genes (*CCD7* and *CCD8*) altogether and an editing efficiency (90%) for a single gene. In our study, callus lines originating from individual transfected protoplasts (Cas9: sgRNA2 for *DcACO1* and cas9:sgRNA1 for *DcACS1*) were selected to assess targeted indels after an 8 week culture period. The 75% *DcACO1*-edited callus exhibited the presence of short indels, predominantly featuring a + 1 bp insertion. In contrast, 25% showed a deletion of—8 bp. Of the detected callus lines, 75% were monoallelic mutants, and 25% were biallelic mutants. Similarly, of the *DcACS1*-edited callus lines, 60% showed short indels, primarily featuring a + 1 bp insertion, while 40% exhibited larger insertions (+ 9 or + 11 bp). This finding aligns with the observations made by [40], who similarly identified mutations with varying patterns in callus lines derived from protoplast-derived calli of canola. The in-frame mutations identified within the *DcACO1* and *DcACS1* genes have the potential to induce modifications or partial impairments to the functionality of their respective encoded proteins. The protein sequences resulting from the CRISPR/Cas9 edits within both genes were examined in a subsequent analysis. Our findings showed deviations in these sequences when compared to the wild-type protein sequence. The specific nature of these deviations was dependent on the specific type of indel. This observation provides empirical support for the hereditary transfer of mutations within the callus lines, resulting in a complete loss of functionality in the *DcACO1* and *DcACS1* genes. To the best of our knowledge, the present report provides the first data for precise editing of the carnation genome using CRISPR/Cas9 RNPs.

## Conclusion

Our study has demonstrated the effectiveness of CRISPR/Cas9 tool in achieving targeted mutagenesis within the *DcACO1* and *DcACS1* gene loci in carnation protoplasts. Our results revealed that the mutation efficiency rates varied depending on the specific sgRNAs designed for these gene loci. As the CRISPR RNP method facilitated editing of the *DcACO1* and *DcACS1* without introducing foreign DNA, this approach offers several advantages, particularly in situations where GMO regulations do not apply, thereby enhancing the efficiency and cost-effectiveness of CRISPR-Cas9 technology for crop enhancement. It sets the stage for the development of novel floricultural crops with desirable traits, thereby contributing significantly to the advancement of floricultural industry.

### Supplementary Information

**Additional file 1: Table S1.** sgRNAs sequence used for carnation *DcACO1* and *DcACS1* gene editing, **Table S2.** List of primers used for PCR and sequencing, **Table S3.** PGR combinations and concentration used for protoplast culture, **Figure S1.** List of sgRNA generated by Rgen Cas-designer tool, **Figure S2.** Schematic flow chart of the cleavage assay, **Figure S3.** Targeted deep sequencing analysis of transformed carnation protoplasts for *DcACO1* and *DcACS1* genes.

## Data Availability

Data is contained within the article or Additional file materials.
