# Supplementary material for: Editing of the ethylene biosynthesis gene in carnation using CRISPR-Cas9 ribonucleoprotein complex
Source: Plant Methods. 2024 Feb 2;20:20. doi: 10.1186/s13007-024-01143-0 (PMC10835871; doi:10.1186/s13007-024-01143-0)
Supplement: Supplementary file 1 — Additional file 1: Table S1. sgRNAs sequence used for carnation DcACO1 and DcACS1 gene editing, Table S2. List of primers used for PCR and sequencing, Table S3. PGR combinations and concentration used for protoplast culture, Figure S1. List of sgRNA generated by Rgen Cas-designer tool, Figure S2. Schematic flow chart of the cleavage assay, Figure S3. Targeted deep sequencing analysis of transformed carnation protoplasts for DcACO1 and DcACS1 genes. [file 13007_2024_1143_MOESM1_ESM.docx]

**Additional file 1: Table S1.** sgRNAs sequence used for carnation *DcACO1* and *DcACS1* gene editing.

| Gene | Name | Sequence  ‘5 ˗ 3’ | Length | PAM |
| --- | --- | --- | --- | --- |
| *DcACO1* | g1 | TAGCGCAGACAGATGGTAAC | 20 | AGG |
|  | g2 | CATCTGTCTGCGCTATCACG | 20 | CGG |
| *DcACS1* | g1 | CATGGGCATGCCCGGCTTTA | 20 | GGG |
|  | g2 | CCAGCATTTCACAAGCGAGC | 20 | TGG |
|  | g3 | CGCGGCATTGTTTGTTTGGA | 20 | TGG |
|  | g4 | CCTTCCTGTGCTCTGAGCCA | 20 | GGG |
|  | g5 | CTCTGAGCCAGGGTGGTTTA | 20 | GGG |

**Additional file 1: Table S2.** List of primers used for PCR and sequencing.

| **Name** | **Sequence 5’˗3’** | **Expected Product**  **length (bp)** | **Rounds** | **Purpose** |
| --- | --- | --- | --- | --- |
| **ACO1_F_1**  **ACO1_R_1** | AACATCTCCGAGGTCCCTGA  TGAGATGAGATGAGAGTGGCG | 852 | PCR | - |
| **ACO1_F_2**  **ACO1_R_2** | GAAGCCGCCTCCGACTAATA  CAAACCTGGGCTCCTTCTCT | 325 | 2^nd^ round PCR | Sequencing  for sgRNA 1 and 2 |
| **ACS1_F_1**  **ACS1_R_1** | ATGACATGCAGATTCCGCGA  ACCCTTCCACGGGTTACAAA | 1217 | PCR | - |
| **ACS1_F1_2**  **ACS1_R1_2** | GTCACAAACCCGTCAAATCCC  TCGACATTCGACGAGCAGTT | 315 | 2^nd^ round PCR | Sequencing  for sgRNA 1 |
| **ACS1_F2_2**  **ACS1_R2_2** | AAATCCTGGGTACGTCACTGC  GGTCGCATTGTCCATGTTGG | 280 | 2^nd^ round PCR | Sequencing for sgRNA 2, 3, and 5 |

**Additional file 1: Table S3.** PGR combinations and concentration used for protoplast culture.

| **No** | **PGR (mg/l)** |
| --- | --- |
| 1 | 1.0 Zeatin |
| 2 | 0.5 2,4-D |
| 3 | 1.0 2,4-D |
| 4 | 0.5 NAA |
| 5 | 0.5 NAA 0.5 2,4-D |
| 6 | 0.5 Zeatin, 1.0 2,4-D |
| 7 | 1.0 Zeatin, 1.0 2,4-D |
| 8 | 2.0 Zeatin, 1.0 2,4-D |
| 9 | 0.5 BA, 1.0 2,4-D |
| 10 | 1.0 BA, 1.0 2,4-D |
| 11 | 0.5 TDZ, 1.0 2,4-D |
| 12 | 1.0 TDZ, 1.0 2,4-D |
| 13 | 1.0 Zeatin, 1.0 NAA |
| 14 | 1.0 Zeatin, 3.0 NAA |
| 15 | 1.0 Zeatin, 5.0 NAA |

**a.**

**b.**

**Additional file 1: Figure S1.** List of sgRNA generated by Rgen Cas-designer tool*.* Selected sgRNAs are highlighted in colours. **a.** *DcACO1* Green: sgRNA1, Red: sgRNA2. **b.** *DcACS1* Green: sgRNA1, Red: sgRNA2, Yellow: sgRNA3, Blue: sgRNA4, Purple: sgRNA5


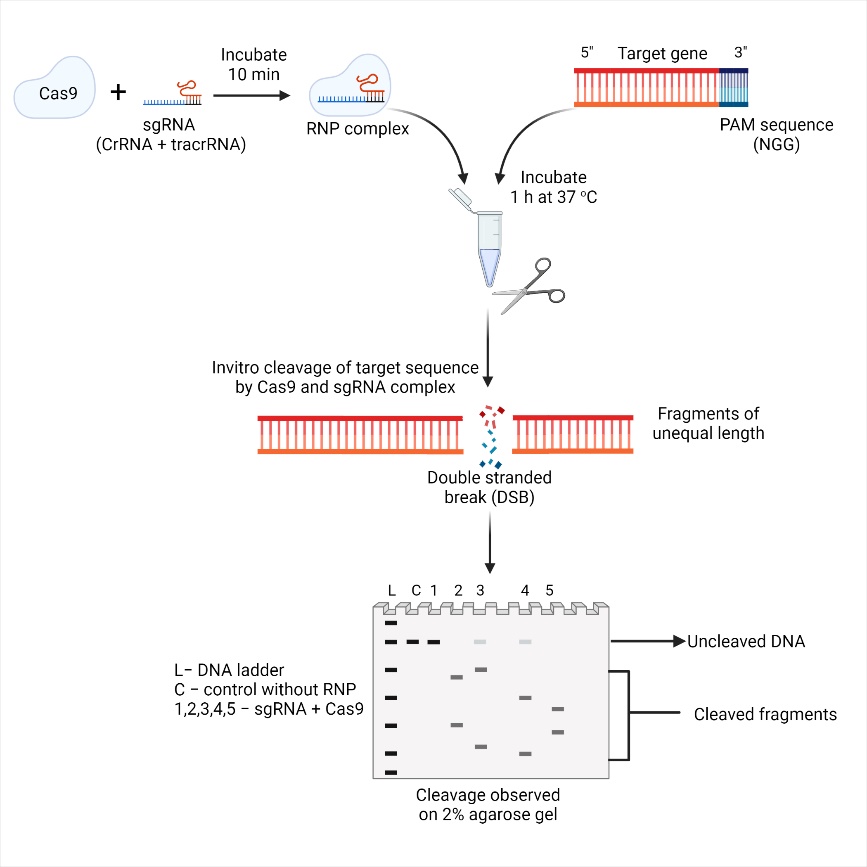


**Additional file 1: Figure S2.** Schematic flow chart of the cleavage assay, created with [biorender.com](http://www.biorender.com)

**Additional file 1: Figure S3.** Targeted deep sequencing analysis of transformed carnation protoplasts for *DcACO1* and *DcACS1* genes showing the complete indels and mutation patterns. Blue- Target sequence, red- PAM sequence, green- insertions, orange- substitution.
